# Point-of-care screening for hepatitis B virus infection in pregnant women at an antenatal clinic: A South African experience

**DOI:** 10.1371/journal.pone.0181267

**Published:** 2017-07-21

**Authors:** Nafiisah Chotun, Wolfgang Preiser, Christoffel Johannes van Rensburg, Pedro Fernandez, Gerhard Barnard Theron, Dieter Glebe, Monique Ingrid Andersson

**Affiliations:** 1 Division of Medical Virology, Department of Pathology, Faculty of Medicine and Health Sciences, Stellenbosch University, Tygerberg, Cape Town, South Africa; 2 National Health Laboratory Service (NHLS), Tygerberg Hospital, Tygerberg, South Africa; 3 Division of Gastroenterology and Hepatology, Department of Medicine, Stellenbosch University, Tygerberg, Cape Town, South Africa; 4 Division of Urology, Department of Surgical Sciences, Stellenbosch University, Tygerberg, Cape Town, South Africa; 5 Department of Obstetrics and Gynaecology, Stellenbosch University, Tygerberg, Cape Town, South Africa; 6 Institute of Medical Virology, National Reference Centre for Hepatitis-B and D Viruses, Justus-Liebig-University Giessen, German Centre for Infection Research (DZIF), Giessen, Germany; 7 Oxford University Hospitals NHS Foundation Trust, Oxford, United Kingdom; University of North Carolina at Chapel Hill School of Dentistry, UNITED STATES

## Abstract

**Background & aims:**

Elimination of HIV and syphilis mother-to-child transmission (MTCT) has received much attention but little consideration has been given to the possibility of elimination of HBV MTCT. In sub-Saharan Africa, HBV vertical transmission continues to be reported and it remains an important public health problem. This study aimed to assess the feasibility of screening pregnant women for HBV using a point-of-care (POC) test and implementing interventions to prevent HBV MTCT.

**Methods:**

In this observational prospective cohort study, HIV-uninfected pregnant women who consented to testing were screened for HBV using a rapid POC test for HBsAg. Positive results were laboratory-confirmed and tested for HBV DNA and serological markers. Women with viral loads ≥ 20 000 IU/ml received tenofovir (TDF) treatment and all infants received birth-dose HBV vaccine. Two blood samples collected six months apart from HBV-exposed infants within their first year of life were tested for HBV DNA.

**Results:**

Of 144 women who were approached, 134 consented to participating (93% acceptance rate of HBV POC test). Six women tested positive for HBsAg (4.5%; 95% CI 0.99%–8.01%), all confirmed by laboratory testing. Two mothers, M1 and M4, were treated with TDF during their third trimester of pregnancy. Six HBV-exposed infants received the HBV vaccine within 24 hours of birth, of whom two were lost to follow-up and four (including the two born to M1 and M4) had undetectable levels of HBV DNA when tested at the two time points.

**Conclusion:**

We found that HBV screening using POC testing fulfilled the criteria considered necessary for implementation. It has acceptable performance, is inexpensive, reliable, and was well accepted by the study participants. Screening pregnant women as part of the HBV MTCT prevention strategy is therefore feasible in a South African clinical setting.

## Introduction

Hepatitis B virus (HBV) infection causes substantial, and largely unappreciated, morbidity and mortality. Globally, it is estimated that 248 million people have an active HBV infection [1] and that every year 686 000 people die as a direct consequence of this infection [2]. This is despite the availability of a safe and effective vaccine and potent antiviral therapy.

Acute HBV infection in the immunocompetent adult is likely to be cleared. However, perinatal transmission of HBV in neonates and horizontal transmission in young children carry a high risk of chronicity, which can result in serious complications such as liver cirrhosis and hepatocellular carcinoma. Around 90% of those infected at delivery will become chronic carriers, whilst less than 5% of adults will develop chronic infections [3]. Efforts to control ongoing infection with HBV should, therefore, be focused on preventing infection in the neonate [4].

The World Health Organization (WHO) recommends administration of the first dose of the hepatitis B (HB) vaccine within 24 hours of delivery [5]. There are perceived difficulties with the implementation of this recommendation [6]. Many pregnant women do not have access to the vaccine because there are no national guidelines or policies on the use of the birth-dose vaccine, they do not deliver in healthcare facilities, and with the rollout of the hexavalent vaccine (DTaP-IPV-HB-Hib) in South Africa, vaccination against HBV at birth would need to be administered as a single separate vaccine, adding cost to the schedule.

Furthermore, infants of mothers who are HBeAg positive or who have high HB viral loads are at higher risk of perinatal infection than women who have lower viral loads. A meta-analysis investigating the risk of perinatal transmission of HBV in Sub-Saharan Africa showed that in HBeAg-positive women, the pooled risk of transmission was 38.3% (95% CI: 7.0–74.4%) compared to only 4.8% (95% CI: 0.1–13.3%) in HBeAg-negative women [7]. According to international guidelines, such as the American Association for the Study of Liver Diseases (AASLD) and the European Association for the Study of the Liver (EASL) guidelines, HBV-exposed infants should receive both hepatitis B immunoglobulin (HBIG) and HBV birth-dose vaccine to further reduce the risk of transmission [8,9]. However, HBIG is not available in many resource-poor settings due to its high cost and the logistics of storage and administration. Antiviral therapy during pregnancy reduces the risk of HIV transmission. Nucleoside or nucleotide analogues, for example, lamivudine or tenofovir (TDF), when administered around 28 to 32 weeks of gestation reduce maternal HB viral load during pregnancy and therefore the risk of mother-to-child transmission (MTCT) [10,11]. Antiviral treatment of HBV-infected mothers with high viral loads should be considered in order to reduce the risk of perinatal transmission.

Sub-Saharan Africa (SSA), after Asia, carries the highest burden of HBV infection [1]. Antenatal screening for HBV infection is currently not performed in the public sector in South Africa, but it is the key step in identifying those women most at risk of transmitting infection and allowing the implementation of preventive strategies.

This study was carried out to assess the feasibility of (i) screening pregnant women for hepatitis B surface antigen (HBsAg) using a point of care (POC) test, (ii) offering antiviral treatment to those women with viral loads greater than 20 000 IU/ml since HBIG was not easily available, and (iii) administering HBV birth-dose vaccine to infants of HBV-infected mothers within 24 h of birth, to successfully prevent vertical transmission of HBV in a resource-limited setting.

## Methods

### Study population: Enrolment

This observational prospective cohort study was approved by the Health Research Ethics Committee of Stellenbosch University, Cape Town, South Africa (N13/10/132) and conformed to the 1975 Declaration of Helsinki. From June to November 2014, HIV-uninfected pregnant women already enrolled in an ongoing unrelated study at Tygerberg Hospital, a tertiary care hospital in Cape Town, South Africa, were approached for participation. The study nurse provided an information session on HBV, the study aims, and offered HBV POC testing to potential study participants.

### Maternal HBV testing: Screening, confirmation, and additional markers

The women provided written informed consent before their participation in this study and were counselled and screened for HBsAg by a trained and experienced research nurse. A finger prick blood sample was taken from each participant and tested using the Determine^™^ HBsAg point-of-care test (Alere Inc., MA, USA). This assay was chosen because of its acceptable performance [12]. Additional venous blood samples were collected from those with positive screening test results for confirmatory HBsAg testing and subsequent testing for additional HBV markers (anti-HBc [total and IgM], HBeAg, and anti-HBe) on the ARCHITECT i2000SR automated system (Abbott Laboratories, IL, USA). In addition, these participants had their HBV DNA levels quantified using the automated COBAS^®^ AmpliPrep/COBAS^®^ TaqMan^®^ HBV Test, v2.0 (Roche Molecular Systems, NJ, USA). The limit of quantification of this viral load assay using serum samples was 20 IU/ml. If a patient had a detectable but unquantifiable viral load (i.e., below 20 IU/ml), their result was reported as < 20 IU/ml. However, if the patient had undetectable viral loads, their result was reported as lower than the detectable limit (LDL). In all cases, the viral load results were interpreted in conjunction with the patient’s clinical status, which included the patient’s liver function tests, clinical examination and serological results and whether there was any clinical evidence of liver cirrhosis (clinical signs of chronic liver disease or liver dysfunction observed either on ultrasound or abnormal liver function tests). Genotyping was also performed on DNA extracted from the maternal serum samples using an in-house PCR assay targeting the polymerase/surface region of the viral genome [13].

### Interventions to prevent MTCT of HBV: Treatment and vaccination

International guidelines, such as the AASLD guidelines, do not recommend treating women with HB viral loads lower than 200 000 IU/ml as their infants will be receiving both HBIG and HBV birth-dose vaccine. However, in the present study, since HBIG was not available for use owing to its high cost, treatment was discussed and offered to participating pregnant women with HB viral loads greater than 20 000 IU/ml. Those who qualified for treatment under these criteria were reviewed at the Hepatology Clinic and offered a 300 mg TDF tablet daily, starting after 28 weeks gestation until one month after delivery. The women were monitored for hepatic flares for one month post-treatment. The risks and benefits of therapy were clearly explained. All HBV-exposed infants received a birth dose of the standard monovalent, yeast-derived HBV vaccine (Heberbiovac HB: 10 μg in 0.5 ml). They were subsequently vaccinated with the hexavalent vaccine (Hexaxim^®^ Sanofi Pasteur: 10 μg of HBsAg in 0.5 ml) at 6 and 10 weeks [5].

### Infant HBV testing: Screening and follow-up

A heel prick whole blood sample was collected from each HBV-exposed infant as soon as possible after birth and spotted onto a Guthrie card. The dried blood spots (DBS) thus obtained were eluted overnight in COBAS^®^ AmpliPrep/COBAS^®^ TaqMan^®^ Specimen Pre-Extraction Reagent (SPEX) and tested for HBV DNA using the automated COBAS^®^ AmpliPrep/COBAS^®^ TaqMan^®^ HBV Test, v2.0 (Roche Molecular Systems, NJ, USA). The limit of quantification of this viral load assay using DBS was 1000 IU/ml. If a patient had a detectable but unquantifiable viral load (i.e., below 1000 IU/ml), their result was reported as < 1000 IU/ml. If a patient had an undetectable viral load, their result was reported as lower than the detection limit (LDL). Just as for the adult cases, the viral load results were interpreted in conjunction with the patient’s clinical status and serological results.

A serum sample was collected from HBV-exposed infants seven months after the initial HBV test to determine their HBsAg status and whether any previously positive infant had a persistent, and therefore chronic, HBV infection. This sample was tested for serological markers of HBV infection (HBsAg and anti-HBc IgM) and immunity (anti-HBs) and for HBV DNA and HBV genotype, using the same assays described above for the maternal samples.

## Results

### Study population

The study nurse approached 144 HIV-uninfected pregnant women for this study. Only ten women declined to participate in this study. The reasons provided for not participating were as follows: reticence to be tested for a sexually transmitted disease and inconvenience of coming back to the hospital for follow-up testing for study purposes. A total of 134 women were thus enrolled, giving the HBV POC test a 93% acceptance rate. Their mean age was 26.1 (range: 18–40) years and all were in their second (62/134) or third trimester (72/134) of pregnancy.

### Maternal HBV testing

Of the 134 women, six had reactive HBsAg screening test results (4.5%; 95% CI 0.99%–8.01%). All were confirmed by laboratory-based testing. There were no indeterminate results. Their mean age was 27 (range: 21–34 years). The results from serological and molecular tests performed on the women (coded M1 to M6) are presented in Table 1. Mothers M1, M2, M4, and M6 were infected with HBV subgenotype D3 and mother M5 with HBV subgenotype A1. The HB viral load of mother M3 was too low for sequencing.

**Table 1:** Serological and molecular maternal results.

Mother	HBsAg*	HBeAg	Anti-HBe	Anti-HBc IgM	Anti-HBc (total)	HBV DNA (IU/ml)	HBV genotype
***M1***	+	−	+	−	+	23 000	D3
***M2***	+	−	+	−	+	5120	D3
***M3***	+	−	+	−	+	91	ND^#^
***M4***	+	+	−	−	+	767 000	D3
***M5***	+	−	−	−	+	336	A1
***M6***	+	−	+	−	+	161	D3

IU/ml: International Units per millilitre; HBsAg: hepatitis B surface antigen; HBeAg: hepatitis B envelope antigen; Anti-HBe: antibody to hepatitis B envelope antigen; Anti-HBc: antibody to hepatitis B core antigen; IgM: Immunoglobulin M; HBV: hepatitis B virus; M: HBV-infected mother; −: Negative; +: Positive; ND: Not done.

* HBsAg status was determined by point-of-care testing (Determine^™^, Alere Inc., USA) and confirmed by ELISA (ARCHITECT i2000SR, Abbott, USA).

^**#**^ Sequencing not possible due to low viral load.

### Interventions to prevent MTCT of HBV

Mothers M2, M3, M5, and M6 had low HB viral loads and therefore were considered to be at low risk of transmitting HBV to their infants perinatally [14]. Mothers M1 and M4 had higher HB viral loads and were therefore offered antiviral treatment with TDF at 36 and 28 weeks of pregnancy, respectively. One month after giving birth, mother M1’s treatment was discontinued and she was monitored for any hepatic flares for one additional month. She remained well after stopping TDF. When she was tested one month after giving birth, her viral load was < 20 IU/ml. Mother M4 was not tested post-delivery as she declined to attend her hospital appointment. However, she was contacted telephonically and indicated that she remained well.

All six infants (coded C1 to C6 and born to mothers M1 to M6 respectively) received the first dose of the HBV vaccine at birth and subsequently the second and third doses at ages 6 and 10 weeks, respectively. Although infants C5 and C6 were lost to follow-up, mothers M5 and M6 were contacted telephonically and verbally confirmed taking C5 and C6 for their 6-week and 10-week vaccinations.

### Infant HBV testing

DBS were collected from four of six HBV-exposed infants (C1 to C4) at a mean age of 97 days (range: 34–151 days). Two infants, C5 and C6 born to mothers M5 and M6, were lost to follow-up. The four tested infants, C1 to C4, were all male. All four DBS samples had undetectable levels of DNA. Follow-up serum samples were collected from those four infants seven months after the first sample at a mean age of 328 (274–381) days. All four infants had undetectable levels of HBV DNA and protective levels of anti-HBs (Table 2). They also tested negative for anti-HBc IgM.

**Table 2:** Serological and molecular infant results.

	1^st^ sample (DBS)		2^nd^ sample (serum)			
Child	HBV DNA	HBsAg	Anti-HBc (IgM)	HBsAg	Anti-HBs*	HBV DNA
***C1***	LDL^#^	−	−	−	525	LDL^#^
***C2***	LDL^#^	−	−	−	629	LDL^#^
***C3***	LDL^#^	−	−	−	350	LDL^#^
***C4***	LDL^#^	−	−	−	360	LDL^#^

The DBS were collected at a mean age of 97 days (range: 34–151 days) and the serum at a mean age of 328 (274–381) days. C1 was born to mother M1, C2 to mother M2, C3 to mother M3, and C4 to mother M4.

DBS: Dried blood spot; HBsAg: hepatitis B surface antigen; Anti-HBc: antibody to hepatitis B core antigen; IgM: Immunoglobulin M; Anti-HBs: antibody to hepatitis B surface antigen; HBV: hepatitis B virus; C: HBV-exposed child; −: Negative;

*: Anti-HBs levels measured in mIU/ml.

^#^: LDL—Lower than detectable limit. The viral load was below the detection limit of the assay.

## Discussion

### Feasibility of implementation of HBV POC testing

This study describes the implementation of HBsAg screening in an antenatal clinic in the Western Cape, South Africa. Screening tests typically are required to fulfil the Wilson-Jungner criteria [15] to be considered suitable for implementation. Firstly, the disease being identified (in this case HBV infection) needs to be a major health problem that can be detected in the early stages using the screening test and confirmed using diagnostic tests that should be available and accessible to the tested population. Furthermore, the latest WHO guidelines recommend routinely offering HBsAg testing to pregnant women in settings with a ≥2% HBsAg seroprevalence in the general population [16]. HBV is a serious unrecognised and under-diagnosed public health issue in South Africa, with a reported prevalence as high as 23% in HIV-infected individuals from the Limpopo province [17]. In the current study, the prevalence of HBsAg in HIV-uninfected pregnant women was 4.5%, which is slightly higher than the prevalence of 2.9% which was reported in a previous study on HIV-uninfected pregnant women from the Western Cape Province, South Africa [18]. Although the seroprevalence of HBsAg in the general South African population is currently unknown, it is clear that the HBsAg prevalence observed in the current and previous studies are worrying and justify the introduction of HBV POC testing, particularly for pregnant women. HBV POC testing in pregnant women can identify those who are actively infected and at risk of transmitting HBV to their children. Further diagnostic testing may be performed to better quantify the risk of transmission.

Secondly, treatment for the disease should be available and accessible to the tested population. Of the six women diagnosed with active HBV infection in the present study, two (M1 & M4) had high viral loads and were therefore at high risk of transmitting HBV to their infants. It was interesting to note that four of five sequenced mothers were infected with subgenotype D3 even though the most prevalent genotype in South Africa is A1. They were able to access treatment through the public health care system. Furthermore, all HBV-exposed children in this study received HBV birth-dose vaccine. The combined effect of these interventions was successful in preventing HBV MTCT including in the two high-risk cases. All four infants were negative for HBV markers of infection, including HBsAg and anti-HBc IgM, when tested at two different time points, six months apart.

Thirdly, the test needs to be acceptable to the population. The present study found that HBV POC testing was acceptable to most pregnant women with only a few objecting to being tested (10/144). We believe that if knowledge of the disease and testing were to become more widespread, acceptability would increase further as has been demonstrated previously with HIV counselling and rapid antibody testing [19].

Finally, the test itself needs to be sensitive, specific, easy to use, non-invasive, quick, and cost-effective. This study found that all positive rapid test results were confirmed to be true-positives in the laboratory, giving the test 100% specificity for the present study. We cannot comment on the sensitivity of the test in this study since the negative results were not confirmed; however, previous studies have reported the pooled sensitivity of the test to be 97.6% (95% Credible Interval: 96.3%–98.6%) and the analytical sensitivity of the Determine^™^ HBsAg test to be between 1 and 2 IU/ml [12,20]. We also cannot fully exclude the possibility that false negatives may have occurred as reported elsewhere [21]; however, this is unlikely, since the prevalence of active HBV infection observed in this study was 4.5% (95% CI: 0.99%–8.01%), which is in line with our previous data for a Western Cape maternal population [18]. Although no economic analyses were performed within the present study, a recent community-based study from The Gambia, where the HBsAg prevalence is 8.8%, found that a screen-and-treat intervention would produce an incremental cost-effectiveness ratio (ICER) of $540 per disability-adjusted life year averted, which was considered to be highly cost-effective as it was in line with the WHO’s willingness-to-pay level of once the country’s gross domestic product-per-capita ($487). When the HBsAg prevalence was reduced to 5%, the ICER increased slightly to $633 but was still considered to be cost-effective [22], suggesting that a similar intervention could also be potentially cost-effective in South Africa.

### Feasibility of implementation of HBV POC testing—The South African socio-political context

Several international guidelines have addressed the management of pregnant women with chronic hepatitis B. In Asia, which has the highest burden of HBV worldwide, the Asian Pacific Association for the Study of the Liver (APASL) recommends screening pregnant women during their first trimester so that unprotected mothers can be vaccinated against HBV and HBV-infected mothers can be treated as early as possible if necessary [23]. The AASLD guidelines also strongly recommend treating women with HB viral loads higher than 200 000 IU/ml in the third trimester of pregnancy until delivery and providing both HBIG and HBV birth-dose vaccine to the infant [8]. However, in the present study, HBIG was not accessible and therefore could not be administered to the HBV-exposed infants; an HB viral load of 20 000 IU/ml was therefore selected as a cut-off to offer treatment. Both guidelines recommend the use of TDF as the treatment of choice in pregnant women and recent studies have shown that the use of TDF is safe during pregnancy and breastfeeding and does not affect early childhood development [10,24,25].

The failure in South Africa to screen pregnant women for HBsAg and to vaccinate infants at delivery perpetuates the cycle of infection in high-risk communities. Routine HBV screening can and should be implemented using existing prenatal POC testing infrastructures, alongside HIV and syphilis POC tests, that are already offered to pregnant women in South Africa following the ‘opt-out provider-initiated testing and counselling’ (opt-out PITC) model. In the opt-out PITC model, rapid HBV testing would be offered to all pregnant women presenting at a healthcare facility. The pregnant women could then decide whether to get tested or not. This model has been shown to increase the uptake of HIV testing and this would be especially beneficial for HBV testing as it is currently a largely unknown disease to the general South African public.

For those women who require treatment, TDF is accessible in South Africa, either as the single drug Viread^®^ or in combination form as Truvada^®^. Although in the Western Cape Province Viread^®^ has been prescribed to treat HBV mono-infection through the public and private health care systems, access to the treatment has been notoriously difficult as mono-infected patients in the public health care system could previously only access medication at a tertiary hospital. However, since mid-2016, this access has been extended to local day hospitals. HBV/HIV co-infected patients would preferentially receive Truvada^®^ as it is generally prescribed as first-line therapy in HIV-infected individuals. Unlike Viread^®^, Truvada^®^ is available free of charge at primary health care clinics, making it easier for HIV/HBV co-infected patients to access treatment than HBV mono-infected patients. In 2016, Truvada^®^ was also approved for use in HIV prevention in South Africa and in the context of pre-exposure prophylaxis against HIV, appears that it can also be safely administered to HBV mono-infected individuals [26].

The WHO and a number of major international liver health organisations (e.g. EASL, AASLD, APASL) strongly recommend that all children born to HBV-infected mothers should receive a birth dose of the HBV vaccine to reduce the risk of vertical transmission [8,9,23,27]. Subsequent doses of the HBV vaccine can then be administered as part of the hexavalent (DTaP-IPV-HB-Hib) vaccines that are routinely administered to infants as part of the EPI at 6, 10, and 14 weeks [27]. While there are some structural barriers to the implementation of the HBV birth-dose vaccine, they are not insurmountable [6]. In SSA, many pregnant women do not have access to the vaccine because they do not deliver in healthcare facilities and there is a cold chain requirement for vaccines. However, the HBV vaccine has been shown to be stable outside of the cold chain, making it possible for vaccination to be administered outside of a health care setting [28].

In South Africa, the monovalent HBV vaccine will no longer be supplied to the Expanded Programme on Immunisation (EPI), as the HBV vaccine is incorporated in the newly introduced hexavalent DTaP-IPV-HB-Hib vaccine which is administered at age 6, 10 and 14 weeks. Therefore, HBV-exposed infants urgently needing a birth-dose vaccine will need to receive either an HBV vaccine, which will have to be purchased privately or a half-dose of the adult monovalent vaccine (Heberbiovac HB: 20 μg in 1 ml). This again is not part of the standard EPI repertoire of vaccines and thus not affordable for many families. This development poses an unnecessary additional hurdle to transpose the WHO recommendation for general introduction of an HBV birth-dose vaccine within the South African EPI.

The lack of political will in South Africa has been detrimental to the implementation of HBV health policies. The South African Department of Health needs to intervene and put into place national guidelines that recommend and support introduction of the birth-dose vaccine, screening of pregnant women, and treatment for those who qualify for it. Only then will we be able to begin to control the ongoing cycle of HBV infection in communities in South Africa.

### Limitations

The major limitation of this study was that it was conducted at a tertiary hospital, following recruitment in primary care by a dedicated study team. The feasibility of implementation in a primary care antenatal clinic setting should be addressed in future studies. Furthermore, the sample size was small as this was designed to be a pilot study. A larger study of pregnant women in a primary health care setting is currently underway.

## Conclusions

HBV is an important public health problem that requires more attention. We have shown that screening pregnant women for HBV is feasible in a South African clinical setting. Screening for HBV infection fulfils the Wilson-Jungner criteria. The POC test is reliable, inexpensive, and was readily accepted by the participants of this study. This study also provided some evidence that screening pregnant women, providing antivirals to treat those with high HB viral loads, and providing HBV birth-dose vaccine to HBV-exposed infants can be implemented in South Africa. This data supports the call to South African politicians and policymakers to establish clear guidance on the prevention of HBV MTCT. It is only this which will reduce the perpetual cycle of infection in African communities and enable us to reduce, indeed eliminate this eminently preventable infection.

## Supporting information

S1 Table Data associated with tested mothers. (XLSX)

## References

[1] Schweitzer A, Horn J, Mikolajczyk RT, Krause G, Ott JJ. Estimations of worldwide prevalence of chronic hepatitis B virus infection: a systematic review of data published between 1965 and 2013. Lancet (London, England). 2015;386: 1546–55. doi: 10.1016/S0140-6736(15)61412-X 10.1016/S0140-6736(15)61412-X 26231459

[2] GBD 2013 Mortality and Causes of Death Collaborators. Global, regional, and national age—sex specific all-cause and cause-specific mortality for 240 causes of death, 1990–2013: a systematic analysis for the Global Burden of Disease Study 2013. Lancet. Elsevier Ltd; 2015;385: 117–171. doi: 10.1016/S0140-6736(14)61682-2 25530442 10.1016/S0140-6736(14)61682-2 PMC4340604

[3] Beasley RP, Trepo C, Stevens CE, Szmuness W. The e antigen and vertical transmission of hepatitis B surface antigen. Am J Epidemiol. 1977;105: 94–98. doi: 10.1136/bmj.2.6099.1416-d 835566 10.1093/oxfordjournals.aje.a112370

[4] Andersson MI, Rajbhandari R, Kew MC, Vento S, Preiser W, Hoepelman AIM, et al Mother-to-child transmission of hepatitis B virus in sub-Saharan Africa: time to act. Lancet Glob Heal. Elsevier; 2015;3: e358–e359. doi: 10.1016/S2214-109X(15)00056-X 10.1016/S2214-109X(15)00056-X 26087980

[5] World Health Organization. Hepatitis B vaccines: WHO position paper—Recommendations. Vaccine. 2010;28: 589–590. doi: 10.1016/j.vaccine.2009.10.110 19896455 10.1016/j.vaccine.2009.10.110

[6] World Health Organization. Preventing Perinatal Hepatitis B Virus Transmission: A Guide for Introducing and Strengthening Hepatitis B Birth Dose Vaccination [Internet]. 2015 [cited 15 May 2017] pp. 1–112. http://www.who.int/iris/handle/10665/208278

[7] Keane E, Funk AL, Shimakawa Y. Systematic review with meta-analysis: the risk of mother-to-child transmission of hepatitis B virus infection in sub-Saharan Africa. Aliment Pharmacol Ther. 2016;44: 1005–1017. doi: 10.1111/apt.13795 27630001 10.1111/apt.13795

[8] Terrault NA, Bzowej NH, Chang K-M, Hwang JP, Jonas MM, Murad MH. AASLD guidelines for treatment of chronic hepatitis B. Hepatology. 2016;63: 261–283. doi: 10.1002/hep.28156 26566064 10.1002/hep.28156 PMC5987259

[9] Lampertico P, Agarwal K, Berg T, Buti M, Janssen HLA, Papatheodoridis G, et al EASL 2017 Clinical Practice Guidelines on the management of hepatitis B virus infection. J Hepatol. Epub 2017: 4 18 doi: 10.1016/j.jhep.2017.03.021 28427875 10.1016/j.jhep.2017.03.021

[10] Brown RS, McMahon BJ, Lok ASF, Wong JB, Ahmed AT, Mouchli MA, et al Antiviral therapy in chronic hepatitis B viral infection during pregnancy: A systematic review and meta-analysis. Hepatology. 2016;63: 319–333. doi: 10.1002/hep.28302 26565396 10.1002/hep.28302

[11] Pan CQ, Duan Z, Dai E, Zhang S, Han G, Wang Y, et al Tenofovir to Prevent Hepatitis B Transmission in Mothers with High Viral Load. N Engl J Med. 2016;374: 2324–2334. doi: 10.1056/NEJMoa1508660 27305192 10.1056/NEJMoa1508660

[12] Shivkumar S, Peeling R, Jafari Y, Joseph L, Pai NP. Rapid point-of-care first-line screening tests for hepatitis B infection: a meta-analysis of diagnostic accuracy (1980–2010). Am J Gastroenterol. 2012;107: 1306–1313. doi: 10.1038/ajg.2012.141 22641308 10.1038/ajg.2012.141

[13] Chotun N, Nel E, Cotton MF, Preiser W, Andersson MI. Hepatitis B virus infection in HIV-exposed infants in the Western Cape, South Africa. Vaccine. 2015;33: 4618–4622. doi: 10.1016/j.vaccine.2015.06.076 26163924 10.1016/j.vaccine.2015.06.076

[14] Wen WH, Chang MH, Zhao LL, Ni YH, Hsu HY, Wu JF, et al Mother-to-infant transmission of hepatitis B virus infection: Significance of maternal viral load and strategies for intervention. J Hepatol. 2013;59: 24–30. doi: 10.1016/j.jhep.2013.02.015 23485519 10.1016/j.jhep.2013.02.015

[15] Andermann A, Blancquaert I, Beauchamp S, Déry V. Revisiting Wilson and Jungner in the genomic age: a review of screening criteria over the past 40 years. Bull World Health Organ. 2008;86: 317–9. doi: 10.2471/BLT.07.050112 18438522 10.2471/BLT.07.050112 PMC2647421

[16] World Health Organization. WHO guidelines on hepatitis B and C testing [Internet]. 2017 [cited 15 May 2017]. http://www.who.int/hepatitis/publications/guidelines-hepatitis-c-b-testing/en/

[17] Lukhwareni A, Burnett RJ, Selabe SG, Mzileni MO, Mphahlele MJ. Increased detection of HBV DNA in HBsAg-positive and HBsAg-negative South African HIV/AIDS patients enrolling for highly active antiretroviral therapy at a Tertiary Hospital. J Med Virol. 2009;81: 406–412. doi: 10.1002/jmv.21418 19152393 10.1002/jmv.21418

[18] Andersson MI, Maponga TG, Ijaz S, Barnes J, Theron GB, Meredith SA, et al The epidemiology of hepatitis B virus infection in HIV-infected and HIV-uninfected pregnant women in the Western Cape, South Africa. Vaccine. 2013;31: 5579–5584. doi: 10.1016/j.vaccine.2013.08.028 23973500 10.1016/j.vaccine.2013.08.028 PMC3898695

[19] Wanyenze RK, Nawavvu C, Namale AS, Mayanja B, Bunnell R, Abang B, et al Acceptability of routine HIV counselling and testing, and HIV seroprevalence in Ugandan hospitals. Bull World Health Organ. 2008;86: 302–309. doi: 10.2471/BLT.07.042580 18438519 10.2471/BLT.07.042580 PMC2647415

[20] Servant-Delmas A, Duong LT, Hamon C, Houdah AK, Laperche S. Comparative performance of three rapid HBsAg assays for detection of HBs diagnostic escape mutants in clinical samples. J Clin Microbiol. 2015;53: 3954–3955. doi: 10.1128/JCM.02117-15 26378274 10.1128/JCM.02117-15 PMC4652127

[21] Njai HF, Shimakawa Y, Sanneh B, Ferguson L, Ndow G, Mendy M, et al Validation of rapid point-of-care (POC) tests for detection of hepatitis B surface antigen in field and laboratory settings in the Gambia, Western Africa. J Clin Microbiol. 2015;53: 1156–1163. doi: 10.1128/JCM.02980-14 25631805 10.1128/JCM.02980-14 PMC4365211

[22] Nayagam S, Conteh L, Sicuri E, Shimakawa Y, Suso P, Tamba S, et al Cost-effectiveness of community-based screening and treatment for chronic hepatitis B in The Gambia: an economic modelling analysis. Lancet Glob Heal. 2016;4: e568–e578. doi: 10.1016/S2214-109X(16)30101-2 10.1016/S2214-109X(16)30101-2 27443782

[23] Sarin SK, Kumar M, Lau GK, Abbas Z, Chan HLY, Chen CJ, et al Asian-Pacific clinical practice guidelines on the management of hepatitis B: a 2015 update. Hepatol Int. 2016;10: 1–98. doi: 10.1007/s12072-015-9675-4 26563120 10.1007/s12072-015-9675-4 PMC4722087

[24] M. le Roux S, Jao J, Brittain K, Phillips TK, Olatunbosun S, Ronan A, et al Tenofovir exposure in utero and linear growth in HIV-exposed, uninfected infants. AIDS. 2017;31: 97–104. doi: 10.1097/QAD.0000000000001302 27898591 10.1097/QAD.0000000000001302 PMC5814299

[25] Mofenson LM, Baggaley RC, Mameletzis I. Tenofovir disoproxil fumarate safety for women and their infants during pregnancy and breastfeeding. AIDS. 2017;31: 213–232. doi: 10.1097/QAD.0000000000001313 27831952 10.1097/QAD.0000000000001313

[26] Solomon MM, Schechter M, Liu AY, McManhan VM, Guanira JV. Hance RJ, et al The Safety of Tenofovir—Emtricitabine for HIV Pre-Exposure Prophylaxis (PrEP) in Individuals With Active Hepatitis B. JAIDS J Acquir Immune Defic Syndr. 2016;71: 281–286. doi: 10.1097/QAI.0000000000000857 26413853 10.1097/QAI.0000000000000857 PMC4752387

[27] World Health Organization. Guidelines for the prevention, care and treatment of persons with chronic hepatitis B infection [Internet]. 2015 [cited 15 May 2017] p. 166 http://apps.who.int/iris/bitstream/10665/154590/1/9789241549059_eng.pdf?ua=1&ua=1 26225396

[28] Hipgrave DB, Maynard JE, Biggs BA. Improving birth dose coverage of hepatitis B vaccine. Bull World Health Organ. 2006;84: 65–71. 16501717 10.2471/blt.04.017426 PMC2626514

